# Α Markov model for longitudinal studies with incomplete dichotomous outcomes

**DOI:** 10.1002/pst.1794

**Published:** 2016-12-05

**Authors:** Orestis Efthimiou, Nicky Welton, Myrto Samara, Stefan Leucht, Georgia Salanti, on behalf of GetReal Work Package 4

**Affiliations:** ^1^ Department of Hygiene and EpidemiologyUniversity of Ioannina School of MedicineIoanninaGreece; ^2^ School of Social and Community MedicineUniversity of BristolBristolUK; ^3^ Department of Psychiatry and PsychotherapyTechnische Universität MünchenMunichGermany; ^4^ Institute of Social and Preventive MedicineUniversity of BernBernSwitzerland

**Keywords:** Bayesian analysis, missing data, multistate models

## Abstract

Missing outcome data constitute a serious threat to the validity and precision of inferences from randomized controlled trials. In this paper, we propose the use of a multistate Markov model for the analysis of incomplete individual patient data for a dichotomous outcome reported over a period of time. The model accounts for patients dropping out of the study and also for patients relapsing. The time of each observation is accounted for, and the model allows the estimation of time‐dependent relative treatment effects. We apply our methods to data from a study comparing the effectiveness of 2 pharmacological treatments for schizophrenia. The model jointly estimates the relative efficacy and the dropout rate and also allows for a wide range of clinically interesting inferences to be made. Assumptions about the missingness mechanism and the unobserved outcomes of patients dropping out can be incorporated into the analysis. The presented method constitutes a viable candidate for analyzing longitudinal, incomplete binary data.

## 1 INTRODUCTION

Missing outcome data are frequently encountered in randomized control trials and may compromise the validity of inferences and increase the uncertainty in the effects of an intervention.^1^ Missing data constitute a major problem for certain areas of medicine. Studies in mental health usually have a high dropout rate due to the nature of the conditions and the treatments involved. Studies in schizophrenia tend to have a dropout rates as large as 50% or even higher, which leads to large amounts of missing outcome data.^2^

In the presence of missing data, the researcher can follow a number of different approaches for the analysis. The simplest of all is to analyze only patients that completed the study after excluding patients that dropped out (*complete case analysis*, *CCA*). This approach, however, will lead to a loss of precision and to biased results when data are not *missing completely at random* (*MCAR*).^1^, ^3^, ^4^, ^5^, ^6^ An MCAR mechanism is implausible in many clinical contexts, where dropout rates are informative: In psychiatric trials, for example, dropout is strongly correlated to the clinical outcome and is often considered a proxy to both treatment efficacy and acceptability. A common way to overcome the missing data problem is to employ some form of imputation, for example, the last observation is carried forward (LOCF),^6^ multiple imputations,^6^, ^7^ or to use regression methods that do not impute data such as the mixed model for repeated measures (MMRM)^8^, ^9^ and selection models.^10^ The various available methods make different assumptions about the unobserved outcomes. The missingness mechanism, however, is essentially untestable. Thus, researchers sometimes choose to analyze dropout separately as an additional (and often primary) outcome.

The aim of this paper is to present an alternative method for analyzing incomplete dichotomous outcomes. We do this by employing a Markov model previously proposed, after adapting it for the missing outcome data context. Markov models offer an intuitive approach for modeling patients' transitions between a number of discrete states over time^11^, ^12^, ^13^, ^14^ and have been used in a variety of clinical settings such as modeling terminal and nonterminal events in coronary heart disease,^15^ cancer screening,^16^ and HIV progression.^17^ Markov models can be either discrete time (where transitions can only occur at fixed time points) or continuous time (where transitions can occur at any time).^18^ Continuous‐time Markov models have both theoretical and practical advantages over their discrete‐time counterparts.^18^ The model we use in this paper is a continuous‐time Markov model with 3 states,^19^ and our method is focused on the reconstruction of the various paths a patient may follow across these states. It can be used to model dichotomous, patient‐level outcomes reported over different time points while accounting for patients dropping out and for patients relapsing. To the best of our knowledge, Markov models have not been previously used to analyze missing patient outcomes. The analysis is focused on the estimation of the transition rates between the different states rather than probabilities of transitions which depend on the elapsed time. Using these rates, the model can provide an array of clinically interesting estimates regarding treatments' effects on efficacy, acceptability, and relapse at any time point. We also expand the model by including additional unobserved states so as to accommodate assumptions regarding the missingness mechanism and the outcomes of patients dropping out. We adopt a Bayesian framework throughout this paper because it offers increased flexibility in modeling. We fit all presented models employing Markov chain Monte Carlo (MCMC) techniques in WinBUGS. A frequentist approach is also possible by using for example the msm package in R.^20^

The paper is structured as follows: in section 2, we provide a brief description of the data that motivate our methods. In section 3, we present the model, we discuss how to estimate the model's parameters from the available data, we describe how the model can be used to make various inferences on the relative treatment effects, and we present model extensions. In section 4, we present the results from the application, and in section 5, we discuss the advantages and the limitations of our approach. In Appendix S1, we provide mathematical details, additional results as well as the WinBUGS code that we use to implement the model.

## 2 DATA DESCRIPTION

To exemplify our methods, we use data from a randomized controlled trial comparing amisulpride with risperidone for patients in the acute phase of schizophrenia.^21^ For each patient and for each time point, the study provides information on whether or not the patient is a responder (with response defined as a 50% reduction in the Brief Psychiatric Rating Scale from baseline) or has dropped out of the study. A total of 115 patients were randomized in the amisulpride arm and 113 in the risperidone arm. Patients were followed‐up at 1, 2, 4, 6, and 8 weeks after starting to receive medication. The dropout rates were large for both study arms (31% for amisulpride and 26% for risperidone). Once a patient dropped out of the study, the trialists could not collect any efficacy data. No information was available for the reasons of dropping out. For the purposes of this paper, we coded as dropouts all patients that missed a visit and all subsequent ones. In the dataset, we were given access there were no intermediate missing values; that is, there were no missing observations for patients still in the study. In section 1 of Appendix S1, we provide the aggregated outcome data at each time point.

## 3 METHODS

In this section, we present the model and we discuss how to estimate the model parameters from the available data and how to use these estimates to make inferences about the relative treatment effects.

### 3.1 The 3‐state model

**Figure 1:**
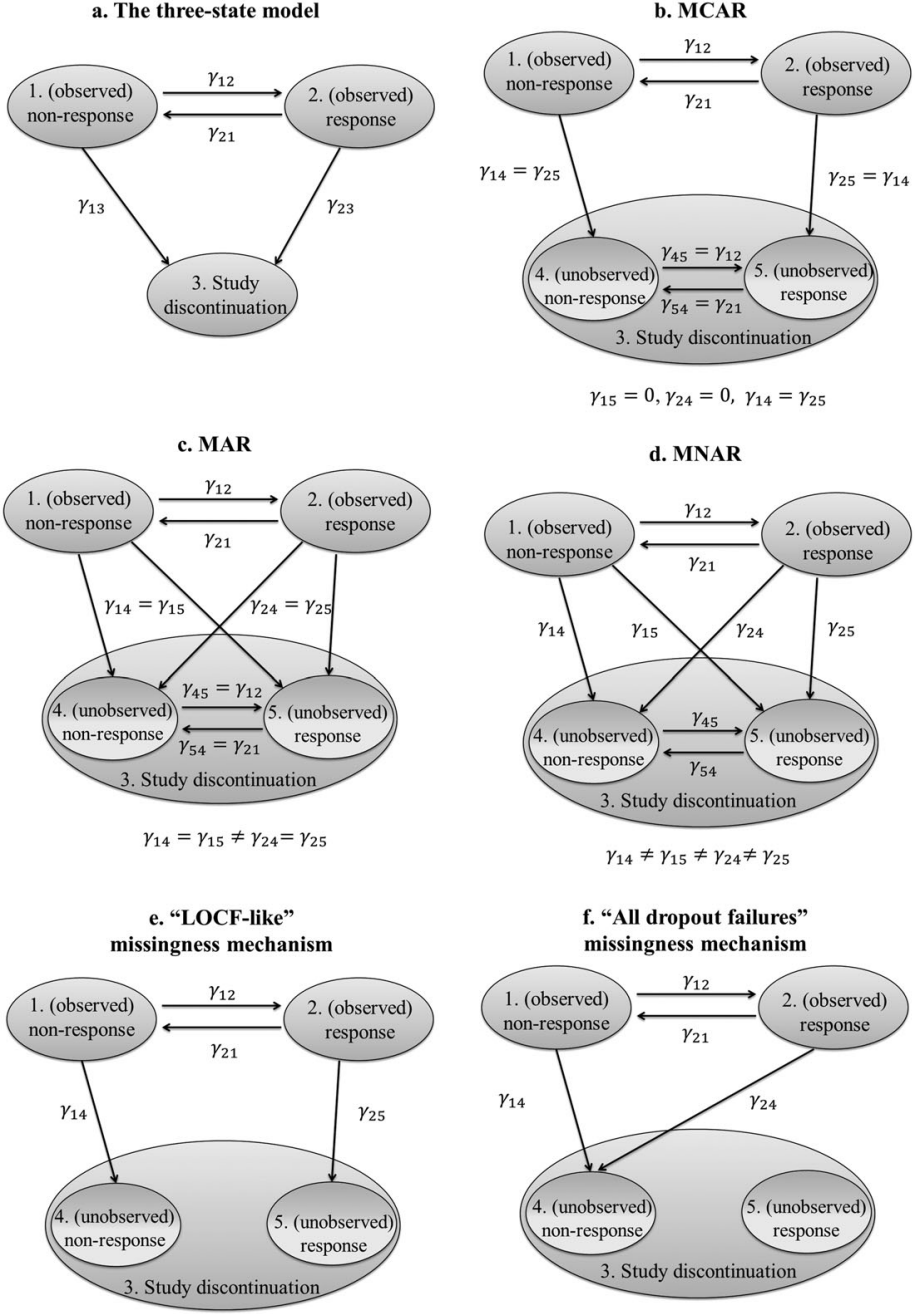
(A) A 3‐state model with transitions between states 1 and 2 allowed both ways. State 3 is an absorbing state and corresponds to a patient dropping out of the study. Each transition from state *Χ* to state *Ψ* is associated with a transition rate *γ*_ΧΨ_. (B‐F) Four‐state models with 2 unobserved states incorporating different assumptions about the missingness mechanism

The basic model that we use comprises 3 different Markov states and is sometimes termed the “illness‐death” model.^14^ State 1 is the starting state for all patients (which we call “[*observed*] *nonresponse*”), state 2 corresponds to a 50% reduction of the score in the Brief Psychiatric Rating Scale from baseline (“[*observed*] *response*”), and state 3 corresponds to the patient dropping out of the study (“*study discontinuation*”). Transitions between states 1 and 2 are allowed in both directions. State 3 is an all‐absorbing state; that is, no backward transitions are allowed and patients dropping out of the study cannot re‐enter. Note, however, that a patient may miss a visit without dropping out of the study; in such cases, the corresponding observation is missing but the patient is not coded in the dropout state. The 3 states of the model and the allowed transitions are depicted in Figure 1A. The 4 *γ* presented in the figure are the target parameters of the model, which we aim to estimate from the available data.

We employ the Markov assumption^14^ that the probability of a transition from a state to another does not depend on the previous states visited or on the time spent in current state, and we also assume that the transition rates *γ*_ΧΨ_ are constant through time and are common for all patients randomized in the same treatment arm. The model can be extended to include patient‐level random effects in the transition rates to make the transition rates dependable on patient‐level covariates (as we discuss in section 3.5) or to account for time‐dependency in the transition rates (see section 5).

For patient *k*, randomized in treatment arm *T*_k_, (*T*_k_ = 1 , 2), the matrix of transition rates is defined as follows:

$$G^{T_{k}}=\left(\begin{array}{ccc} -\left(\gamma_{12}^{T_{k}}+\gamma_{13}^{T_{k}}\right) & \gamma_{12}^{T_{k}} & \gamma_{13}^{T_{k}}\\ \gamma_{21}^{T_{k}} & -\left(\gamma_{21}^{T_{k}}+\gamma_{23}^{T_{k}}\right) & \gamma_{23}^{T_{k}}\\ 0 & 0 & 0 \end{array}\right)$$

Each diagonal element in row *q* (where *q* = 1 , 2 , 3) in the matrix shows the total rate of transition *out*‐*of*‐state *q*. Off‐diagonal elements show how transitions from state *q* are distributed to all other states. For example, $G_{11}^{T_{k}}=-\left(\gamma_{12}^{T_{k}}+\gamma_{13}^{T_{k}}\right)$, which means that patients leave state 1 at a rate of $\gamma_{12}^{T_{k}}+\gamma_{13}^{T_{k}}$ and these patients either go to state 2 (with a transition rate equal to $G_{12}^{T_{k}}=\gamma_{12}^{T_{k}}$) or to state 3 (with a transition rate equal to $G_{13}^{T_{k}}=\gamma_{13}^{T_{k}}$). Because the state‐space is exhaustive, the total number of patients in the system remains constant, and each row of the $G^{T_{k}}$ matrix sums to 0. State 3 is an all‐absorbing state, so the elements of the third row are all 0.

The matrix of transitions, $G^{T_{k}}$, is related to the matrix of the transition probabilities $\Pi^{T_{k}}\left(\Delta ,\text{t}\right)$,

$$\Pi^{T_{k}}(\Delta \text{t})=\left(\begin{array}{ccc} \pi_{1,1}^{T_{k}}\left(\Delta ,\text{t}\right) & \pi_{1,2}^{T_{k}}\left(\Delta ,\text{t}\right) & \pi_{1,3}^{T_{k}}\left(\Delta ,\text{t}\right)\\ \pi_{2,1}^{T_{k}}\left(\Delta ,\text{t}\right) & \pi_{2,2}^{T_{k}}\left(\Delta ,\text{t}\right) & \pi_{2,3}^{T_{k}}\left(\Delta ,\text{t}\right)\\ 0 & 0 & 1 \end{array}\right).$$

The *ΧΨ* element of this matrix gives the probability of a patient receiving treatment *T*_k_, starting at state *Χ* to be found at state *Ψ* after time *Δt*. So, a patient situated at state 2 after time *Δt* will be at states 1, 2, or 3, with probabilities $\pi_{2,1}^{T_{k}}\left(\mathrm{\Delta t}\right)$, $\pi_{2,2}^{T_{k}}\left(\mathrm{\Delta t}\right)$, and $\pi_{2,3}^{T_{k}}\left(\mathrm{\Delta t}\right)$, respectively; of course, these probabilities must add up to 1. A patient situated at state 3 will remain there, so that $\pi_{3,1}^{T_{k}}\left(\mathrm{\Delta t}\right)$ and $\pi_{3,2}^{T_{k}}\left(\mathrm{\Delta t}\right)$ are 0, while $\pi_{3,3}^{T_{k}}\left(\mathrm{\Delta t}\right)$ equals 1 for all time points.

The transition probability matrix $\Pi^{T_{k}}\left(\Delta ,\text{t}\right)$ can be calculated by using the transition rate matrix $G^{T_{k}}$ via Kolmogorov forward equation $\frac{d\Pi^{T_{k}}\left(\Delta ,\text{t}\right)}{d\left(\Delta ,\text{t}\right)}=\Pi^{T_{k}}\left(\Delta ,\text{t}\right)\;G^{T_{k}}$. This is a differential equation giving the evolution of $\Pi^{T_{k}}$ over time. Under regularity conditions which are readily satisfied in practice (and which regard the derivatives of the probability functions^22^), the solution is given by $\Pi^{T_{k}}\left(\Delta ,\text{t}\right)=e^{\Delta \text{tGTk}}=\sum_{n=0}^{\infty }\frac{\Delta t^{n}}{n!}{\left(G^{T_{k}}\right)}^{n}$. Because $G^{T_{k}}$ is a matrix, a closed‐form solution cannot be always obtained. For the special case of a 3‐state model presented in Figure 1A, analytic closed‐form solutions are available.^19^ We first define the $h^{T_{k}}$ quantities as:

(1) $$h^{T_{k}}=\sqrt{{\left({\lambda_{1}}^{T_{k}}-{\lambda_{2}}^{T_{k}}\right)}^{2}+4\gamma_{12}^{T_{k}}\;\gamma_{21}^{T_{k}},\lambda_{X}^{T_{k}}=\sum \psi \neq x\gamma_{\mathrm{X\psi}}^{T_{K}}}$$

where $\lambda_{Χ}^{T_{k}}\;$is the transition rate from state *Χ* to all other states. The transition probabilities are given by the following equations (after dropping the *T*_*k*_ index denoting treatment arm for simplicity):

(2) $$\pi_{1,1}\left(\mathrm{\Delta t}\right)=\frac{\left(-\lambda_{1}+\lambda_{2}+h\right)e^{-\frac{1}{2}\left(\lambda_{1}+\lambda_{2}-h\right)\mathrm{\Delta t}}+\left(\lambda_{1}-\lambda_{2}+h\right)e^{-\frac{1}{2}\left(\lambda_{1}+\lambda_{2}+h\right)\mathrm{\Delta t}}}{2h}$$

(3) $$\pi_{1,2}\left(\mathrm{\Delta t}\right)=\frac{\left(-\lambda_{1}+\lambda_{2}+h\right)\left(\lambda_{1}-\lambda_{2}+h\right)\left(e^{-\frac{1}{2}\left(\lambda_{1}+\lambda_{2}-h\right)\mathrm{\Delta t}}-e^{-\frac{1}{2}\left(\lambda_{1}+\lambda_{2}+h\right)\mathrm{\Delta t}}\right)}{4h\gamma_{21}}$$

(4) $$\pi_{1,3}\left(\mathrm{\Delta t}\right)=1-\pi_{1,1}\left(\mathrm{\Delta t}\right)-\pi_{1,2}\left(\mathrm{\Delta t}\right)$$

(5) $$\pi_{2,1}\left(\mathrm{\Delta t}\right)=\frac{\gamma_{21}\left(e^{-\frac{1}{2}\left(\lambda_{1}+\lambda_{2}-h\right)\mathrm{\Delta t}}-e^{-\frac{1}{2}\left(\lambda_{1}+\lambda_{2}+h\right)\mathrm{\Delta t}}\right)}{h}$$

(6) $$\pi_{2,2}\left(\mathrm{\Delta t}\right)=\frac{\left(\lambda_{1}-\lambda_{2}+h\right)e^{-\frac{1}{2}\left(\lambda_{1}+\lambda_{2}-h\right)\mathrm{\Delta t}}+\left({-\lambda }_{1}+\lambda_{2}+h\right)e^{-\frac{1}{2}\left(\lambda_{1}+\lambda_{2}+h\right)\mathrm{\Delta t}}}{2h}$$

(7) $$\pi_{2,3}\left(\mathrm{\Delta t}\right)=1-\pi_{2,1}\left(\mathrm{\Delta t}\right)-\pi_{2,2}\left(\mathrm{\Delta t}\right)$$

Given the 4 transition rates, all transition probabilities included in the ${\text{Π}}^{T_{k}}$ matrix can be computed as a function of time. In what follows, we will see how to use the available data to estimate the 4 parameters of the model (the 4 transition rates) and how to make inferences on relative treatment effects. Before this, we introduce some notation that considerably simplifies the analysis.

### 3.2 Data notation

Let us denote *t*_1_, *t*_2_ … *t*_F_ as the time points at which the observations for each patient are collected. To each patient *k*, randomized in treatment arm *T*_k_, and for each time point *t*_m_ (*m* = 1 , 2 ,  … *F*), corresponds an observation of his state, $S_{m}^{k}$ (1, 2, or 3). We also code the patient's state by using a vector $x_{m}^{k}$. This vector conveys the same information as $S_{m}^{k}$ and takes values (1, 0, 0) for state 1 (observed nonresponse), (0, 1, 0) for state 2 (observed response), and (0, 0, 1) for state 3 (study discontinuation). All patients start from the nonresponse state, so that $x_{0}^{k}=\left(1,\;0,\;0\right)\forall i,k.$ These definitions are summarized in Table 1. In section 2 of Appendix S1, we provide a list of all notations used in this paper.

**Table 1:** Data for Patient *k*

Time point	*t*_0_ = 0	*t*_1_	*t*_2_	*t*_F_
State $S_{m}^{k}$ at time *t*_m_	1	2	1	3
	Observed nonresponse	Observed response	Observed nonresponse	Study discontinuation
Data coded as vectors	$x_{0}^{k}=\left(\begin{array}{ccc} 1, & 0, & 0 \end{array}\right)$	$x_{1}^{k}=\left(\begin{array}{ccc} 0, & 1, & 0 \end{array}\right)$	$x_{2}^{k}=\left(\begin{array}{ccc} 1, & 0, & 0 \end{array}\right)$	$x_{F}^{k}=\left(\begin{array}{ccc} 0, & 0, & 1 \end{array}\right)$
Observed transition	–	1 → 2	2 → 1	$S_{F-1}^{k}\to 3$
Transition probabilities being informed by the observed transition	–	$\pi_{1,1}^{T_{k}}\left(\mathrm{\Delta t}\right)$	$\pi_{2,1}^{T_{k}}\left(\mathrm{\Delta t}\right)$	$\pi_{S_{F-1}^{k},1}^{T_{k}}\left(\mathrm{\Delta t}\right)$
		$\pi_{1,2}^{T_{k}}\left(\mathrm{\Delta t}\right)$	$\pi_{2,2}^{T_{k}}\left(\mathrm{\Delta t}\right)$	$\pi_{S_{F-1}^{k},2}^{T_{k}}\left(\mathrm{\Delta t}\right)$
		$\pi_{1,3}^{T_{k}}\left(\mathrm{\Delta t}\right)$	$\pi_{2,3}^{T_{k}}\left(\mathrm{\Delta t}\right)$	$\pi_{S_{F-1}^{k},3}^{T_{k}}\left(\mathrm{\Delta t}\right)$

The numbers show an example where a patient responds to treatment at time point *t*_1_, relapses to nonresponse at time point *t*_2_, and has left the study at time point *t*_F_ *.* Each transition informs the corresponding set of probabilities and consequently the transition rates.

### 3.3 Estimating the model parameters

Our target is to estimate the transition rates from the transition probabilities that are directly estimable from the data. A transition from a starting state $S_{m}^{k}$ (where the patient was observed to be at time *t*_m_) is controlled by the transition probabilities $\pi_{S_{m}^{k},1}^{T_{k}}\left(\mathrm{\Delta t}\right),$ $\pi_{S_{m}^{k},2}^{T_{k}}\left(\mathrm{\Delta t}\right)$, and $\pi_{S_{m}^{k},3}^{T_{k}}\left(\mathrm{\Delta t}\right)$ given in Equations (2) to (7), where *Δt* = *t*_m + 1_ − *t*_m_ corresponds to the time interval between the 2 observations. In the example presented in Table 1, a patient was situated at state 1 at time *t*_0_ (so that $S_{0}^{k}=1$) and he was observed to be at state 2 in the next observation at time *t*_1_ ( $S_{1}^{k}=2$). This corresponds to a transition 1 → 2 in time *Δt* = *t*_1_ − *t*_0_. This transition provides information about the corresponding transition probabilities ${\pi_{S_{0}^{k},1}^{T_{k}}\left(\mathrm{\Delta t}\right)=\pi }_{1,1}^{T_{k}}\left(\mathrm{\Delta t}\right)$, ${\pi_{S_{0}^{k},2}^{T_{k}}\left(\mathrm{\Delta t}\right)=\pi }_{1,2}^{T_{k}}\left(\mathrm{\Delta t}\right)$, and ${\pi_{S_{0}^{k},3}^{T_{k}}\left(\mathrm{\Delta t}\right)=\pi }_{1,3}^{T_{k}}\left(\mathrm{\Delta t}\right)$ and informs the estimation of the 4 transition rates. Likewise, the second transition is a relapse (transition 2 → 1); the corresponding time is *Δt* = *t*_2_ − *t*_1_, and it informs the probabilities ${\pi_{S_{1}^{k},1}^{T_{k}}\left(\mathrm{\Delta t}\right)=\pi }_{2,1}^{T_{k}}\left(\mathrm{\Delta t}\right)$, ${\pi_{S_{1}^{k},2}^{T_{k}}\left(\mathrm{\Delta t}\right)=\pi }_{2,2}^{T_{k}}\left(\mathrm{\Delta t}\right)$, and ${\pi_{S_{1}^{k},3}^{T_{k}}\left(\mathrm{\Delta t}\right)=\pi }_{2,3}^{T_{k}}\left(\mathrm{\Delta t}\right)$, which in turn give information about the 4 transition rates *γ*. Note here that a patient remaining at the same state for 2 consecutive observations also informs the corresponding probabilities. Also note that if a patient misses a visit without dropping out of the study (i.e., data on at least 1 subsequent visit is available), the likelihood is informed by the remaining observations and their corresponding *Δt*. No further assumption is needed to employ the model in such a case.

In summary, each observed transition provides information about 3 of 6 transition probabilities included in ${\text{Π}}^{T_{k}}\left(\mathrm{\Delta t}\right)$. These 3 probabilities are selected for each transition $S_{m}^{k}\to S_{m+1}^{k}$ according to the starting state ( $S_{m}^{k}$) and can be written as the vector ( $\pi_{S_{m}^{k},1}^{T_{k}}\left(\mathrm{\Delta t}\right),\pi_{S_{m}^{k},2}^{T_{k}}\left(\mathrm{\Delta t}\right),\pi_{S_{m}^{k},3}^{T_{k}}\left(\mathrm{\Delta t}\right))={x_{m}^{k}∙\Pi }^{T_{k}}\left(\mathrm{\Delta t}\right)$. The likelihood for the observation on patient *k*, treatment *T*_k_, at the (*m* + 1) time point is given by a multinomial distribution:

(8) $$x_{m+1}^{k}∼\text{Multinomial}\left({x_{m}^{k}∙\Pi }^{T_{k}}\left(\mathrm{\Delta t}\right);1\right)$$

The full likelihood of the data can be obtained as

$$\;L=\prod_{k=1}^{\mathrm{Np}}\prod_{m=0}^{F-1}f_{M}\left(x_{m+1}^{k};{{\;x}_{m}^{k}∙\Pi }^{T_{k}}\left(\Delta t_{k,m+1}\right);1\right)$$

where *Np* denotes the total number of patients, *f*_*M*_ is the probability mass function of the multinomial distribution, and *Δt*_*k* , *m* + 1_ is the time interval between observations *m* and *m* + 1 for patient *k*. In this paper, we adopt a Bayesian framework by using MCMC software to estimate the parameters of the model. One can also use frequentist methods to maximize this likelihood; see for example Refs [13, 23].

### 3.4 Making inferences on relative treatment effects

Having estimated the transition rates for each treatment arm, we can reuse Equations (2) to (7) to estimate the probabilities of transition from a state to another, for any period of elapsed time. Note that the probability of making a transition between 2 states does not depend on time *t* per se; rather, it depends on the elapsed time interval *Δt* considered. So, for a patient in a given state at time *t*, the probability of the patient being found in any state at time *t* + *Δt* will only depend on the elapsed time *Δt* and not on *t*.

Subsequently, inference on relative treatment effects can be made at any time point deemed to be clinically interesting as $\delta =l\left(\pi_{Χ,\Psi }^{1}\left(\mathrm{\Delta t}\right)\right)-l\left(\pi_{Χ,\Psi }^{2}\left(\mathrm{\Delta t}\right)\right)$, where *l* is a link function. For instance, if *l*(*x*) = *logit*(*x*), *X* = 1, and *Ψ* = 2, then *δ* corresponds to the log odds ratio for efficacy among completers. If *l*(*x*) =  ln (*x*), *X* = 2, and *Ψ* = 3, then we obtain the log relative risk for dropping out after responding. In section 3 of the Appendix, we discuss in detail the relative effect measures that can be obtained by using the probabilities of transitions. If multiple studies are analyzed so as to be included in a meta‐analysis, then it would be appropriate to pool on the log‐rate‐ratio scale. Predictions on probabilities can then be made at any chosen time point.

One advantage of the proposed Markov model is that it can also provide estimates which might be of interest to clinicians and which cannot be obtained by the currently available approaches for analyzing longitudinal data. These include the following:

- The probability for a patient to drop out due to inefficacy. This is defined as the probability to dropout after time *Δt* without ever experiencing a response to the treatment, that is, to drop out directly from the (observed) nonresponse state.
- The probability of a patient to drop out after responding. This is defined as the probability to dropout straight after the (observed) response state.
- The expected time spent (*ETS*) in each state can be a useful estimate as it summarizes the effect of the treatment in an easy‐to‐understand manner.

The formulas for these quantities are presented in section 4 of the Appendix.

Note that we have assumed all 4 transition rates to be treatment‐specific. Depending on the clinical context, it might be desirable to set some of them equal across treatment arms.^24^ For example, a common *γ*_23_ can be assumed for both treatments when the rate at which responders drop out is believed to be independent of the treatment received.

### 3.5 Including random effects and patient‐level covariates

Until now, we have assumed transition rates to be common for all patients within each treatment arm. The model can be extended to include random effects after assuming that the transition rates for patients randomized in a treatment arm are not fixed but exchangeable, that is, coming from a common distribution.

More specifically, the logarithm of the transition rate for $\gamma_{Χ\Psi }^{k}$ for patient *k* can be assumed to follow a normal distribution, $\ln\left(\gamma_{Χ\Psi }^{k}\right)∼N{\left(\ln\left(\gamma_{Χ\Psi }^{T_{k}}\right),(\tau_{Χ\Psi }^{T_{k}}\right)}^{2})$, where $\tau_{Χ\Psi }^{T_{k}}$ is the standard deviation of the random effects for the *ΧΨ* transition rate for treatment *T*_k_. Various modeling assumptions can be made about the random effects structure; for example, one can include random effects in some or all of the transitions rates, and the model can be further simplified by allowing a common *τ*^2^ for all transition rates across all treatments.

Covariates can also be easily included in the model by setting $\ln\left(\gamma_{Χ\Psi }^{k}\right)∼N{\left(\ln\left(\gamma_{Χ\Psi }^{T_{k}}\right)+b_{Χ\Psi }^{T_{k}}C^{k},(\tau_{Χ\Psi }^{T_{k}}\right)}^{2})$, where *C*^k^ is a patient‐level covariate for patient *k*. In this equation, $\gamma_{\mathrm{Χ\Psi}}^{T_{k}}$ corresponds to the average, treatment‐specific transition rate centered at 0 value of the covariate *C*^k^. One can follow different approaches to model the coefficient *b*, such as to assume a common value for some of them (e.g., it might be reasonable to set $b_{13}^{T_{k}}=b_{23}^{T_{k}}$) or to assume common coefficients across treatments (to allow for a *b*_ΧΨ_ instead of $b_{\mathrm{Χ\Psi}}^{T_{k}}$); choices should be dictated by the clinical context at hand and after taking into account expert clinical opinion.

With these changes, all probabilities of transitions presented in the analyses of the previous sections now also depend on patient covariates (i.e., we need to write $\pi_{1,2}^{k}$ instead of $\pi_{1,2}^{T_{k}}$), but the rest of the analysis remains unchanged. Adding more covariates is also straightforward.

Note that the model without patient‐level random effects and covariates is a population‐averaged approach; that is, it does not distinguish observations belonging to the same or different individuals. By including random effects and/or covariates in the model, we allow for patient‐specific terms in the analysis.

The general 3‐state model assumes that the 2 dropout rates *γ*_13_ and *γ*_23_ may be different, and thus the transition into the dropout state depends on the current state of the patient. This corresponds to a *missing at random* (*MAR*) assumption; that is, dropout is dependent on observed data (state of the patient). Missing at random is also assumed when including covariates for *γ*_13_ and *γ*_23_ in the analysis. In that case, dropout depends also on (observed) patient characteristics. For an MCAR assumption, one would need to set *γ*_13_ = *γ*_23_ and to exclude any covariates from the analysis. In that case, the dropout rate does not depend on whether or not a patient has responded to the treatment or any other observable or unobservable characteristics. In the following section, we discuss how to expand the model to also accommodate an MNAR assumption and how to use the presented framework to make predictions on unobserved outcomes.

### 3.6 Modeling unobserved response

The analysis presented so far essentially treats response and dropout state as 2 mutually exclusive outcomes. However, it is often of interest to researchers to make inferences about treatment effects in patients that drop out. Moreover, it has been recommended to perform sensitivity analyses regarding the unobserved data to assess the robustness of findings under different scenarios regarding the missingness mechanism.^9^, ^25^

One can extend the method we have presented so far by assuming that patients who drop out continue to undergo transitions between an *unobserved nonresponse* and an *unobserved response* state. Several adaptations of such a 4‐state model are depicted in Figure 1B to F. The quantity of interest is now the probability of either observed or unobserved response, that is, $\pi_{1,2}^{T_{k}}+\pi_{1,5}^{T_{k}}$.

In section 6 of the Appendix, we provide the analytic formulas needed to calculate the transition probabilities for the general 4‐state model, given the 8 transition rates (*γ*_12_ , *γ*_21_ , *γ*_14_ , *γ*_15_ , *γ*_24_ , *γ*_25_ , *γ*_45_ , *γ*_54_). These transition rates, however, cannot be directly estimated from the observed data; unobserved data (the outcomes of dropout patients) would also be needed for fitting this model. Thus, to use the 4‐state model, one needs to first fit the 3‐state model of Figure 1A and estimate the corresponding transition rates. At the second stage, one can use the 4‐state model (after making assumptions regarding the missingness mechanism) to make predictions about the outcome in the patients who have dropped out. Several scenarios are discussed below:

1. Missing completely at random can be modeled by setting *γ*_14_ = *γ*_25_, *γ*_24_ = *γ*_15_ = 0, and also *γ*_45_ = *γ*_12_ and *γ*_54_ = *γ*_21_ (Figure 1B). The first 2 of these equations impose the assumption that the dropout rates do not depend on either observed or unobserved data, and that they are equal among responders and nonresponders. The latter 2 of these equations imply that the transitions between the unobserved response and nonresponse states follow the same pattern as the transitions of patients still in the study. Essentially, these 5 equations imply that dropout and response are 2 independent procedures. Note that for an MCAR assumption, the transition rates *γ*_14_ and *γ*_25_ need to be independent of any covariates.
2. Setting *γ*_14_ = *γ*_15_≠*γ*_24_ = *γ*_25_ corresponds to dropout rates being different across responders and nonresponders but to only depend on observed data (MAR). This is depicted in Figure 1C. Again, one should also assume that *γ*_45_ = *γ*_12_ and *γ*_54_ = *γ*_21_, implying that unseen transitions can be predicted based on the observed data.
3. In a missing not at random (MNAR) scenario (Figure 1D), the dropout rates depend on both observed and unobserved outcomes, and the unobserved outcomes cannot be predicted solely by using the observed data. This can be modeled by setting *γ*_14_ ≠ *γ*_15_ and *γ*_24_ ≠ *γ*_25_. With this formulation, the dropout rate depends on both observed data (state before dropping out) as well as the unobserved data (state after dropping out). The transition rates between the unobserved states can then be assumed to be equal to the corresponding observed ones, that is, *γ*_45_ = *γ*_12_ and *γ*_54_ = *γ*_21_, or researchers might adopt different scenarios to better reflect beliefs about the response patterns of dropout patients.
4. The “LOCF‐like” missingness scenario: All dropouts remain in the last observed state. This can be accomplished in the 4‐state scenario by setting *γ*_15_ = *γ*_24_ = *γ*_45_ = *γ*_54_ = 0. Unlike the usual LOCF approach, however, estimation of transition rates and probabilities uses all available observations and not just the last observation from each patient. This is depicted in Figure 1E. Note that for LOCF, MCAR is necessary, but not sufficient assumption.^9^
5. The “All dropout failure” scenario: this can be achieved by setting *γ*_14_ = *γ*_24_ = 0 and is depicted in Figure 1F. This scenario may be of interest to apply in only one of the treatment arms. For example, patients receiving placebo may be expected to drop out only due to lack of effectiveness (but not due to adverse events).

Note that the 4‐state model can account for uncertainty regarding the missing values by using a stochastic approach to model the patient trajectories after dropping out (this holds for scenarios 1, 2, and 3 above but not for scenarios 4 and 5 which do not model uncertainty in the dropout patients).

Regarding how to choose between these models, we think that the choice should be primarily dictated by the research question, the medical context, and the plausibility of the assumptions that it involves. Different models use different assumptions regarding the dropout mechanism. In a situation where, for example, dropout is thought to be irrelevant to the outcome, model 1 will be sufficient. If, on the other hand, dropout is mainly due to inefficacy, an MNAR assumption, such as in model 3, will be more realistic. Measures of model fit and simulation studies might also help in deciding among models employing similar assumptions.

One of the most popular approaches for analyzing repeated observations of a dichotomous outcome is to employ some form of MMRM. For the case of a binary outcome, a logistic link function is commonly used. An example of an MMRM with random time trends is the following:

(9) $$\text{logit}\left(\pi^{k,j}\right)=\beta_{0}+\beta_{1}t_{j}+\beta_{2}T_{k}+u_{k}t_{j}+\varepsilon_{\mathrm{kj}}$$

In this model, *π*^k , j^ denotes the probability of response for patient *k*, at time *t*_j_; it corresponds to *π*_1 , 2_ + *π*_1 , 5_ for the 4‐state model. *T*_k_ denotes the treatment received (assumed here to be a binary covariate); *u*_k_ is a random, subject‐specific slope; and *ε*_kj_ is the residual. Residuals are assumed to be correlated for each patient across time points, that is, ***ε***_**k**_ ~ *N*(0, ***Σ***), with ***Σ*** being a variance‐covariance matrix that can be estimated from the data. This model can be expanded by adding higher order terms of *t*_j_, treatment‐time interaction terms, patient‐level covariates, or by assuming a structure on ***Σ***. Other link functions could be used instead of logit as long as they map (0, 1) to (−∞, ∞).

Instead of using an arbitrarily chosen link function to model the time dependency of the probability of response, in this paper, we have used a method that models the underlying mechanism of disease progression, starting from elementary concepts such as the transition rates. The corresponding expressions, for example, Equation (2), are somewhat similar to Equation (9), in the sense that they are both exponential functions of *t*.

## 4 ANALYSIS OF THE SCHIZOPHRENIA DATA

In this section, we apply our methods for the study described in section 2. We consider the Markov assumption to be a useful approximation for this example; that is, we assume that the patients' disease progression and dropout rates are only affected by their current state.

### 4.1 Model implementation

We fit our model by WinBUGS; the code can be found in section 7 of the Appendix. We assume minimally informative prior distributions for the logarithm of the *γ*s in each arm, $\ln\left(\gamma_{\mathrm{Χ\Psi}}^{i}\right)∼\text{dunif}\left(-10,5\right)$. These limits are chosen arbitrarily, for estimation reasons.^24^ Because these refer to a logarithmic scale, including bigger or smaller values corresponds to extremely big and small transition rates which may hinder convergence; in practice, changing these limits has little impact on the results. We assume random effects on the log‐transition rates as described in section 3.5, assuming a common heterogeneity *τ* ~ dunif(0, 1). A burn‐in period of 20 000 iterations was used for the MCMC simulation. All statistics that we present in the next section were obtained from the posterior distributions of the parameters, based on 2 independent chains with 20 000 iterations each. Convergence was confirmed by using the Brooks‐Gelman‐Rubin criterion.^26^

### 4.2 Results

We first fit the 3‐state model depicted in Figure 1A, assuming *γ*_13_ ≠ *γ*_23_. We present the estimates of the 4 transition rates for each treatment in Table 2, and we also give the relative treatment effects of the log transition rate ratios. The heterogeneity standard deviation for the log‐transition rates (assumed common) was estimated to be 0.64 (95% credible interval [CrI] 0.31 to 0.92).

**Table 2:** Median Estimates and 95% Credible Intervals (CrI) for the Transition Rates and the Relative Treatment Effects Regarding the Log Transition Rate Ratios for the 3‐State Model

	Amisulpride	Risperidone		
	Median	95% CrI	Median	95% CrI
γ_12_	0.189	[0.143; 0.248]	0.136	[0.100; 0.180]
γ_13_	0.052	[0.032; 0.077]	0.047	[0.030; 0.070]
γ_21_	0.076	[0.042; 0.127]	0.056	[0.028; 0.196]
γ_23_	0.024	[0.009; 0.049]	0.009	[0.002; 0.027]
Transition rate ratios				
	Median	95% CrI		
$\gamma_{12}^{\mathrm{Ris}}/\gamma_{12}^{\mathrm{Ami}}$	0.72	[0.48; 1.07]		
$\gamma_{13}^{\mathrm{Ris}}/\gamma_{13}^{\mathrm{Ami}}$	0.92	[0.51; 1.64]		
$\gamma_{21}^{\mathrm{Ris}}/\gamma_{21}^{\mathrm{Ami}}$	0.73	[0.31; 1.62]		
$\gamma_{23}^{\mathrm{Ris}}/\gamma_{23}^{\mathrm{Ami}}$	0.39	[0.06; 1.83]		

An interesting observation is that all rates are higher in the amisulpride than in the risperidone arm. This is because overall, the patients in the amisulpride arm were observed to make a larger number of transitions; this might imply that all effects of amisulpride (both beneficial and harmful) take place relatively more quickly than that of risperidone. Another interesting (although expected) result is that both dropout rates are much higher for nonresponders than for responders (*γ*_13_ > *γ*_23_): Patients not getting well tend to leave the study at higher rates regardless of the treatment they receive, another indication that dropout is related to response.

**Figure 2:**
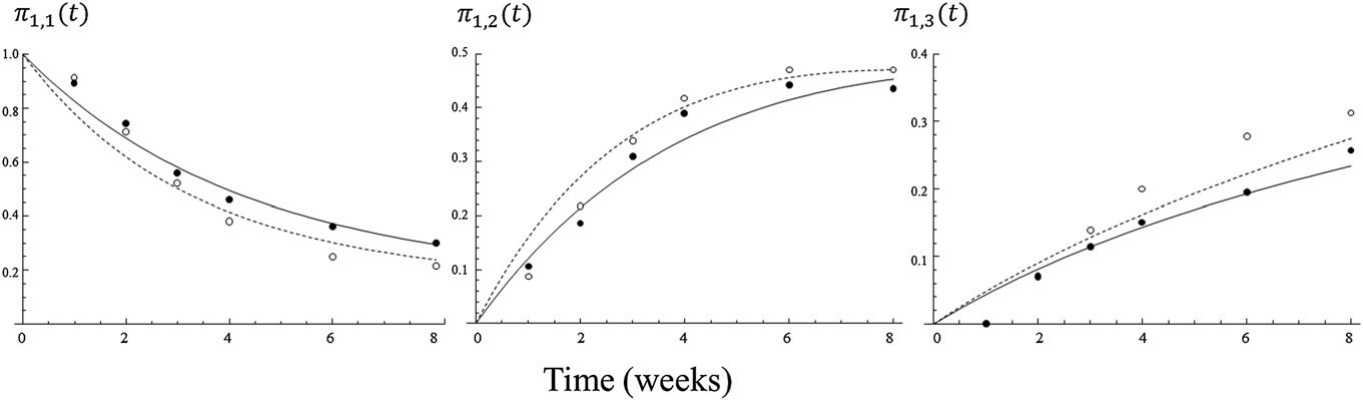
Model estimates from the 3‐state model (lines) and actual observations in the data (dots). The *y*‐axis shows the probability of a patient being found in each state as a function of time (shown on the *x*‐axis). Dashed lines and white dots for amisulpride, thick lines and black dots for risperidone

Figure 2 depicts the fit of the model; the lines correspond to the 3‐state model estimates for the probabilities of transition as a function of time, obtained from Equations (2) to (4); dots correspond to the actual proportion of patients found at each state at each time point in the study. Given that all patients start at state 1 at time 0, these observed proportions correspond to estimates for *π*_11_ , *π*_12_, and *π*_13_. In section 5 of the Appendix, we provide graphs for the time dependency of all transition probabilities. In Table 3, we present various measures estimated from the 3‐state model:

- *OR*_13_ for dropout (*π*_1 , 3_) at study's endpoint (8 weeks).
- *OR*_12_ for (observed) response at study's endpoint (8 weeks), calculated by using *π*_1 , 2_ for each arm.
- *OR*_23_ for a responder to dropout within 8 weeks after responding. This is calculated by using *π*_2 , 3_(*Δt* = 8) for each arm.
- *OR*_21_, for a responder to be found in the nonresponse state 8 weeks after responding. This is calculated by using *π*_2 , 1_(*Δt* = 8) for each arm.
- Expected time spent (*ETS*_X_) on each state *X* for each treatment for the duration of the study.

**Table 3:** Model Estimates for Various Relative Treatment Effects at Study's Endpoint (8 weeks)

Model used	Measure		Median (95% CrI)
3‐state model	*OR*_13_ (values <1 favor risperidone)		0.81 [0.45; 1.50]
	*OR*_12_ (values <1 favor amisulpride)		0.93 [0.54; 1.60]
	*OR*_23_ (values <1 favor risperidone)		0.48 [0.17; 1.41]
	*OR*_21_ (values <1 favor risperidone)		0.96 [0.42; 2.15]
	ETS_1_ Expected time of stay is state 1 (weeks)	Amisulpride	4.0 [3.5; 4.5]
		Risperidone	4.5 [4.0; 5.0]
	ETS_2_ Expected time of stay is state 2 (weeks)	Amisulpride	2.8 [2.2; 3.3]
		Risperidone	2.4 [1.9; 3.0]
	ETS_3_ Expected time of stay is state 3 (weeks)	Amisulpride	1.2 [0.8; 1.7]
		Risperidone	1.1 [0.7; 1.5]
4‐state model	$OR_{\text{response}}^{\text{MCAR}}$ (values <1 favor amisulpride)		0.76 [0.42; 1.38]
	$OR_{\text{response}}^{\mathrm{MAR}}$ (values <1 favor amisulpride)		0.82 [0.47; 1.41]
	$OR_{\text{response}}^{\text{MNAR}}$ (values <1 favor amisulpride)		0.93 [0.53; 1.50]
MMRM	$OR_{\text{response}}^{\text{MMRM}}$ (values <1 favor amisulpride)		0.83 [0.35; 1.96]
CCA	$OR_{\text{response}}^{\mathrm{CCA}}$ (values <1 favor amisulpride)		0.68 [0.35; 1.30]
LOCF	$OR_{\text{response}}^{\text{LOCF}}$ (values <1 favor amisulpride)		0.78 [0.47; 1.32]

All odds ratios are for risperidone (numerator) versus amisulpride (denominator).

According to the median estimates presented in Table 3, amisulpride is only slightly better than risperidone in response among completers (*OR*_12_ = 0.94, 95% CrI 0.54 to 1.60), even though *γ*_12_ is considerably higher for amisulpride; this is because both *γ*_21_ and *γ*_23_ are higher for this drug, so that responders tend to relapse and drop out more often. Regarding dropout, the median estimate is *OR*_13_ = 0.81 (95% CrI 0.45 to 1.50), suggesting risperidone to be slightly better, that is, associated with a smaller probability of study discontinuation. Regarding responders dropping out, the model estimates an *OR*_23_ = 0.48 (CrI 0.17 to 1.41), with values smaller than 1 corresponding to amisulpride being associated with higher probability of a responder dropping out. Finally, there is no notable difference among the treatments regarding relapse, *OR*_21_ = 0.96 (CrI 0.42 to 2.15). Note that all of these estimates correspond to 8 weeks after responding. If deemed clinically interesting, then the model can provide estimates at different time points and the time dependency of the relative treatment effects can also be plotted.

Using the *ETS* in each state for each treatment, we can also calculate percentages of stay in each state. Patients on amisulpride spend on average 50% of the time in nonresponse, 35% in response and 15% of the study. Patients in the risperidone arm spend on average 56% of the time in the nonresponse state, 30% in the response and 14% to the study‐discontinuation state.

We also use the various versions of the 4‐state model depicted in Figure 1 to estimate the following:

- $OR_{\text{response}}^{\text{MCAR}}$ for the probability of either observed or unobserved response (*π*_1 , 2_ + *π*_1 , 5_) at the study's endpoint (8 weeks), under the MCAR scenario. We first fit the 3‐state model setting *γ*_13_ = *γ*_23_, and we estimate the transition rates *γ*_12_ , *γ*_21_, and *γ*_13_ = *γ*_23_. We then use these estimates to calculate the probabilities of transition for the 4‐state model after setting *γ*_14_ = *γ*_25_=*γ*_13_, *γ*_54_ = *γ*_21_, and *γ*_24_ = *γ*_15_ = 0.
- $OR_{\text{response}}^{\mathrm{MAR}}$ for observed or unobserved response (*π*_1 , 2_ + *π*_1 , 5_) at the study's endpoint (8 weeks), under the MAR scenario of section 3.6. We first fit the 3‐state model by assuming *γ*_13_ ≠ *γ*_23_ and then use the 4‐state model, setting *γ*_14_ = *γ*_15_ = 0.5 *γ*_13_, *γ*_24_ = *γ*_25_ = 0.5 *γ*_23_, and *γ*_45_ = *γ*_12_, *γ*_54_ = *γ*_21_.
- $OR_{\text{response}}^{\text{MNAR}}$ for either observed or unobserved response (*π*_1 , 2_ + *π*_1 , 5_) at study's endpoint (8 weeks), under an MNAR scenario. We first fit the 3‐state model, and we then assume *γ*_14_ = 0.9*γ*_13_, *γ*_15_ = 0.1*γ*_13_, *γ*_24_ = 0.9*γ*_23_, *γ*_15_ = 0.1*γ*_23_, *γ*_45_ = 0.1*γ*_12_, and *γ*_54_ = 2*γ*_21_. These choices of the parameters correspond to a scenario where 90% of patients that drop out of the study go to the unobserved nonresponse state and where dropout patients tend to stay to the nonresponse state.

The results from these scenarios are presented in Table 3. We also include in this table the estimate for $OR_{\text{response}}^{\text{MMRM}}$ corresponding to the MMRM described by Equation (9), as well as the LOCF and the CCA estimates.

The results show considerable differences among the various models regarding point estimates. When we adopted an MNAR mechanism, assuming that dropout patients were more probable to end up in nonresponse for both treatments, the OR was closer to 1, as expected. Regarding precision, the LOCF and CCA analyses showed the narrowest confidence intervals. This was also anticipated, because these 2 approaches do not include any uncertainty regarding the unobserved data. The MAR approach of the 4‐state model gave a point estimate similar to the one obtained by using the MMRM (which also assumes MAR). However, the 4‐state model showed a considerable increase in precision compared with MMRM.

To summarize, we conclude that the model suggests amisulpride to be associated with a slightly higher response (even though the difference is not statistically significant). This effect is less pronounced under the illustrative MNAR scenario. Moreover, amisulpride patients tend to drop out more. Relapsing probability has been found to be comparable across treatments.

## 5 DISCUSSION

This paper addresses the problem of missing data in randomized control studies by introducing a method based on multistate Markov models. Our method allows a variety of clinically interesting relative effect measures to be estimated. It can be used to analyze individual participant data on a binary outcome reported at multiple time points, taking into account patients dropping out of the study prematurely as well as information on patients relapsing.

The approach presented here is different than the methods usually applied in the literature for analyzing datasets with missing outcomes. It is focused on modeling the paths patients follow and estimates the probability associated with each path. All estimates follow from a small number of parameters, the transition rates. Assumptions on the unobserved outcomes of patients dropping out can be included in the model on a second stage, after estimating the model's parameters. This is achieved by introducing a set of hidden states as we discussed in section 3.6. Using this 4‐state model, one can readily model the missingness mechanism and incorporate assumptions about unobserved responses of patients that dropped out of the study.

We believe that the proposed method offers considerable advantages compared with currently available methods for handling missing outcomes. First, it can provide a wide range of clinically interesting effect measures and give estimates on their time dependency. The 4‐state model allows for a great flexibility in modeling dropout. In addition, the correlations between all effect measures are automatically incorporated in the model and can be easily estimated by using the code we used for the analysis, which we provide in the Appendix. This is particularly valuable for the case of a meta‐analysis or a network meta‐analysis of these measures. When these correlations are not reported—currently the case in most studies^27^, ^28^—meta‐analysts need to resort to complex modeling to correctly account for them.^29^, ^30^, ^31^, ^32^, ^33^ Also, when studies have different durations, our method can be used to estimate treatment effects for each study at a predefined time before pooling them together in a meta‐analysis.

Our approach is based on the Markov assumption, which implies that the future path of a patient depends only on the present state. This assumption may be more plausible in disease areas where past states of response or nonresponse are expected to have small carry‐over effects on future disease process. Moreover, it might be a suitable assumption to make for the case of chronic conditions, where patients undergo transitions between health states, for example, movements among depressive, manic, and normal states in patients with bipolar disorder. Clinical judgment should be used to assess the plausibility of this assumption depending on the medical context. Note that the Markov assumption can be relaxed if deemed necessary by using several methods; for a discussion, see Ref 34. For example, instead of assuming that patients relapse from the response state back to nonresponse, it might be appropriate to include an additional relapse state in the model. This would account for different future trajectories depending on whether or not patients had responded in the past. Also, we assumed that transition rates remain constant over time, which might be unrealistic for certain clinical conditions, especially for trials with long follow‐up. When this is the case, the proposed methodology can be extended to allow for piecewise constant rates within smaller time periods. In our illustrative example, we could relax the assumption of constant rates to be within time strata, for example, corresponding to first week, 1 to 2 weeks, 2 to 4, 4 to 6, and 6 to 8 weeks. Including different transition rates within each stratum will, of course, improve the fit of the model to the data, at the expense of model complexity. In real‐life practical applications, researchers might want to explore the trade‐off between fit of the model and complexity, using model selection criteria such as deviance information criterion (DIC). Another important limitation of the method we presented is that it requires individual patient data (IPD) to be available. Given that IPD are usually not reported in published studies, if the proposed analysis is not implemented at study level, then it will be hard to use it within a meta‐analysis.

We followed a Bayesian approach to model fitting, and we used WinBUGS. This choice allows for increased flexibility in modeling, but this does not come without a cost: The code we present in the Appendix is computationally intensive and might be slow to converge, especially for very large studies and when multiple covariates are included in the model. In principle, one could instead estimate the model parameters by using maximum likelihood estimate,^13^, ^23^ for example, using the msm package in R^20^; however, further applied research is needed before the Bayesian implementation of the models presented in this paper can be compared with their frequentist counterparts.

The method presented in this paper could be extended to allow for additional states, if this is supported by the data. Let us assume, for example, that a study provides information on the reason of dropping out for each patient: due to side effects, due to lack of efficacy, or due to other reasons. For this case, a 5‐state model, with 3 distinct out‐of‐the‐study states, could be used. One should keep in mind, however, that adding states will, in general, lead to models that cannot be analytically solved by using Kolmogorov forward equation. In this case, a numerical solution can be performed instead,^19^ using WinBUGS Differential Interface, available from http://winbugs‐development.mrc‐bsu.cam.ac.uk/wbdiff.html.

An additional measure that can be calculated by using the methods presented in this paper is the mean quality‐adjusted duration in each state.^35^, ^36^ This can be computed from the *ETS* for each state after weighting them by a quality rate associated with the burden or desirability of each state. Another possible extension to the methodology presented here is about combining results from multiple studies in a meta‐analysis (or a network meta‐analysis when trials comparing different sets of competing treatments are available). Price et al. propose a meta‐analytical framework for Markov models based on parameterizing relative treatment effects regarding log transition rates instead of probabilities.^24^ It would be interesting to explore methods for synthesizing information from studies for which IPD are available together with information coming from studies providing only aggregated data into a single, joint meta‐analysis. Finally, a simulation study would be a very useful follow‐up to the work presented in this paper, as it would allow the evaluation of the performance of the presented methods in comparison with alternative, established methods for analyzing incomplete dichotomous data.

## FUNDING

OE and GS received funding from the Innovative Medicines Initiative Joint Undertaking under grant agreement no 115546, resources of which are composed of financial contribution from the European Union's Seventh Framework Programme (FP7/2007‐2013) and EFPIA companies in kind contribution. The research leading to these results was conducted as part of the GetReal consortium. For further information, please refer to www.imi‐getreal.eu. NW was supported by the UK MRC ConDuCT‐II Hub for Trial Methodology Research (MR/K025643/1). This paper only reflects the personal views of the stated authors.

## Supporting information

Supporting info item
