# Supplementary material for: Α Markov model for longitudinal studies with incomplete dichotomous outcomes
Source: Pharm Stat. 2016 Dec 5;16(2):122–32. doi: 10.1002/pst.1794 (PMC5363348; doi:10.1002/pst.1794)
Supplement: Supplementary file 1 — Supporting info item [file PST-16-122-s001.docx]

# A Markov model for longitudinal studies with incomplete dichotomous outcomes - Appendix

Orestis Efthimiou^[[1]](#footnote-1)^, Nicky Welton^[[2]](#footnote-2)^, Myrto Samara^3^, Stefan Leucht^[[3]](#footnote-3)^, Georgia Salanti^1,4^ on behalf of GetReal Work Package 4

# Aggregated data

The number of patients allocated in each state for each time point is presented in the following table.

|  | **Amisulpride** | | | **Risperidone** | | |
| --- | --- | --- | --- | --- | --- | --- |
|  | non-response | response | dropout | non-response | response | dropout |
| week 1 | 105 | 10 | 0 | 101 | 12 | 0 |
| week 2 | 82 | 25 | 8 | 84 | 21 | 8 |
| week 3 | 60 | 39 | 16 | 63 | 35 | 15 |
| week 4 | 44 | 48 | 23 | 52 | 44 | 17 |
| week 6 | 29 | 54 | 32 | 41 | 50 | 22 |
| week 8 | 25 | 54 | 36 | 34 | 50 | 29 |

# Table of all notations used in the paper

| **Parameter** | **Name** | **Explanation** |
| --- | --- | --- |
| $T_{k}$ | Treatment arm | The treatment received by patient $k$ |
| $\gamma_{X\Psi}^{T_{k}}$ | Transition rates | The transition rate from state $X$ to state $\Psi$ for patient $k$, randomized in treatment arm $T_{k}$ |
| $\boldsymbol{G}^{\boldsymbol{T}_{\boldsymbol{k}}}$ | Transition rates’ matrix | The matrix of all transition rates in the model, assumed common for all patients in each treatment arm |
| $\pi_{X,\Psi}^{T_{k}}(\Delta t)$ | Transition probabilities | The probability that a patient randomized in arm $T_{k}$, observed at state X at time $t$, to be at state $\Psi$ at time $t+\Delta t$. Can be written as a function of the transition rates and $\Delta t$. |
| $\boldsymbol{\Pi}^{\boldsymbol{T}_{\boldsymbol{k}}}\left( \Delta t \right)$ | Transition probabilities’ matrix | The matrix of all transition probabilities for treatment arm $T_{k}$as functions of time. |
| $t_{m}$ | Observation time | The time corresponding to the $m^{th}$observation.  $t_{0}=0$, corresponding to the begging of the study.  $t_{F}$ corresponds to the end of the study. |
| $S_{m}^{k}$ | State | The state at which patient $k$ was observed at the $m^{th}$ observation |
| $\boldsymbol{x}_{\boldsymbol{m}}^{\boldsymbol{k}}$ | Observations coded as vector | The $m^{th}$ observation on patient $k$.  If $\boldsymbol{x}_{\boldsymbol{m}}^{\boldsymbol{k}}=(1,0,0)$ the patient was found to be in the non-response state at time $t_{m}$.  If $\boldsymbol{x}_{\boldsymbol{m}}^{\boldsymbol{k}}=(0,1,0)$ the patient was found to be in the response state at time $t_{m}$  If $\boldsymbol{x}_{\boldsymbol{m}}^{\boldsymbol{k}}=(0,0,1)$ the patient was found to be in the study-discontinuation state at time $t_{m}$ |

# Relative effect measures for the 3-state model

In Section 3.4 of the main paper we discuss the relative effect measures that can be obtained using the probabilities of transitions, Equations (2) – (7) of the main paper. As discussed, these effect measures can be coded as $\delta=l\left( \pi_{X,\Psi}^{1}(\Delta t) \right)-l\left( \pi_{X,\Psi}^{2}(\Delta t) \right)$. For the case that $l$ is the $logit$ function we obtain relative treatment effects in terms of log odds ratios. In the following table we give a brief description of the corresponding relative effect measure.

| $\boldsymbol{l}$ | $\boldsymbol{X,\Psi}$ | **Description of the corresponding measure** |
| --- | --- | --- |
| $logit$ | 1, 1 | $logOR_{11}$: Log odds ratio for patients being non-responders at time zero to be non-responders (and in the study) at time $\Delta t$ |
|  | 1, 2 | $logOR_{12}$ : Log odds ratio for patients being non-responders at time zero to be responders (and in the study) at time $\Delta t$ |
|  | 1, 3 | $logOR_{13}$ : Log odds ratio for patients being non-responders at time zero to be out of the study at time $\Delta t$ |
|  | 2, 1 | $logOR_{21}$ : Log odds ratio for patients being responders at time zero to be non-responders (and in the study) at time $\Delta t$ |
|  | 2, 2 | $logOR_{22}$ : Log odds ratio for patients being responders at time zero to be responders (and in the study) at time $\Delta t$ |
|  | 2, 3 | $logOR_{23}$ : Log odds ratio for patients being responders at time zero to be out of the study at time $\Delta t$ |

If $l$ is the $ln$ function, we obtain relative effects in terms of risk ratios. If $l$ is the identity function, we obtain risk differences.

# Additional formulas for the three-state model

In this paragraph we compute the probability of a patient dropping out *directly*, without visiting the response state at any point. We denote this probability as $\pi_{1,3\left( D \right)}$. To simplify notation we do not include a treatment index in any of the formulas. In order to calculate this quantity let us assume that a study arm $N$ patients situated at state 1. After time $t$ the number of patients still in state 1 that will have made no transitions at all is expected to be $N_{1}\left( t \right)=Ne^{-\left( \gamma_{12}+\gamma_{13} \right)t}.$ Given that the transition rate from state 1 to state 3 is $\gamma_{13}$, between times $t$ and $t+dt$ we expect an average of $dN_{1,3(D)}=\gamma_{13}N_{1}\left( t \right)dt$ direct transitions from *non-response* (state 1) to *study-discontinuation* (state 3) to take place. Replacing $N_{1}\left( t \right)$ we get the following equation: $\frac{dN_{1,3(D)}}{dt}=\gamma_{13}Ne^{-\left( \gamma_{12}+\gamma_{13} \right)t}$. By integrating and after identifying $\pi_{1,3\left( D \right)}=N_{1,3(D)}/N$ we get:

$\pi_{1,3\left( D \right)}(t)=\frac{\gamma_{13}}{\gamma_{13}+\gamma_{12}}(1-e^{-\left( \gamma_{13}+\gamma_{12} \right)t})$.

We also provide here the expression for the probability $\pi_{1\left( 2 \right)3}\left( \Delta t \right)$of a patient to drop out after visiting the response state. Note that this includes the probability of passing through the non-response state more than once, i.e. undergoing transitions $1\to2\to3$, but also $1\to2\to1\to2\to3$ etc. This probability can be computed given the transition rates:

$$\pi_{1\left( 2 \right)3}\left( \Delta t \right)=\int_{0}^{\Delta t} \pi_{12}\left( t^{'} \right)\gamma_{23}dt^{'}=\gamma_{23}\left( -\lambda_{1}+\lambda_{2}+h \right)\left( \lambda_{1}-\lambda_{2}+h \right) \times$$

$$\times\frac{\left( \frac{{1-e}^{-\frac{1}{2}\left( \lambda_{1}+\lambda_{2}-h \right)\Delta t}}{\lambda_{1}+\lambda_{2}-h}-\frac{1-e^{-\frac{1}{2}\left( \lambda_{1}+\lambda_{2}+h \right)\Delta t}}{h+\lambda_{1}+\lambda_{2}} \right)}{2h\gamma_{21}}$$

Quantities $h$ and $\lambda$ were introduced in Equation 1 of the main paper.

Next, we provide the expressions needed to calculate the Expected Time Spent ($ETS$) in each state. Let us assume a patient $k$ is randomized in treatment $T_{k}$. We define $I_{X}^{k}(t)$ to be an indicator function of time, equal to 1 if the patient is in state $X$ at time $t$, zero otherwise. In the infinitesimal time interval between $t$ and $t+dt$ the patient will spend $I_{X}^{k}(t)dt$ time situated at state $X$. The total time spent in state $X$ from the beginning of the study until its end (at time $t_{F}$) is given by:

$$T_{X}^{k}=\int_{0}^{t_{F}} I_{X}^{k}(t)dt$$

The expected value of this quantity is the $ETS_{X}^{T_{k}}$.

$$ETS_{X}^{T_{k}}=E\left[ \int_{0}^{t_{F}} I_{X}^{k}(t)dt \right]=\int_{0}^{t_{F}} E\left[ I_{X}^{k}(t) \right]dt$$

$I_{X}^{k}(t)$ will be equal to 1 with probability $\pi_{1,X}^{T_{k}}(t)$ and equal to zero with probability $1-\pi_{1,X}^{T_{k}}(t)$. Therefore:

$$ETS_{X}^{T_{k}}=\int_{0}^{t_{F}} \pi_{1,X}^{T_{k}}\left( t \right)dt$$

This corresponds to the time a patient randomized in treatment $T_{k}$ is expected to spend situated at state $X$ during the whole duration of the study. Note that patients may make transitions in and out of state $X$, $ETS_{X}^{T_{k}}$ measures the total time expected to be spent there.

# Model estimates

In Figure 1 we present the estimates for all probabilities of transitions corresponding to the 3-state model presented in the main paper.

*Figure 1: Model estimates for all probabilities of transition, blue for Amisulpride, red for Risperidone.* $\boldsymbol{\pi}_{\boldsymbol{X,\Psi}}\boldsymbol{(}\boldsymbol{\Delta}\boldsymbol{t)}$ *corresponds to the probability of a patient in state* $\boldsymbol{X}$ *to be found in state* $\boldsymbol{\Psi}$ *after time* $\boldsymbol{\Delta t.}$

**
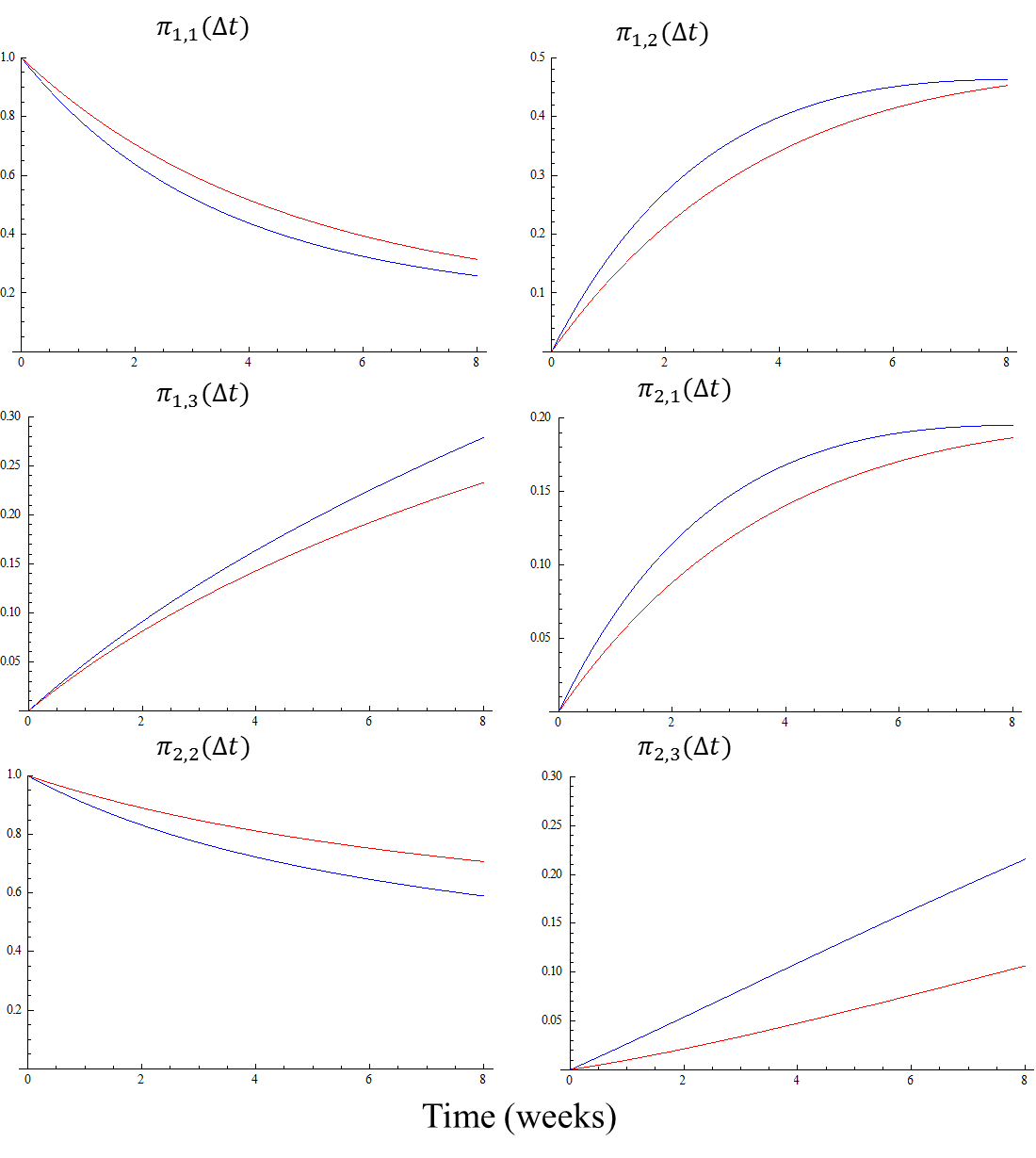
**

In Figure 2 we present the time evolution of various relative effect measures estimated from the model.

*Figure 2: Estimated odds ratios as a function of time* $\boldsymbol{t}$ *since the start of the study (in weeks). The solid lines correspond to the median estimates and the dotted lines denote the 95% Cr.I. An* $\boldsymbol{O}\boldsymbol{R}_{\boldsymbol{12}}$ *larger than 1 favours Risperidone, an* $\boldsymbol{O}\boldsymbol{R}_{\boldsymbol{13}}$ *larger than 1 favours Amisulpride.*


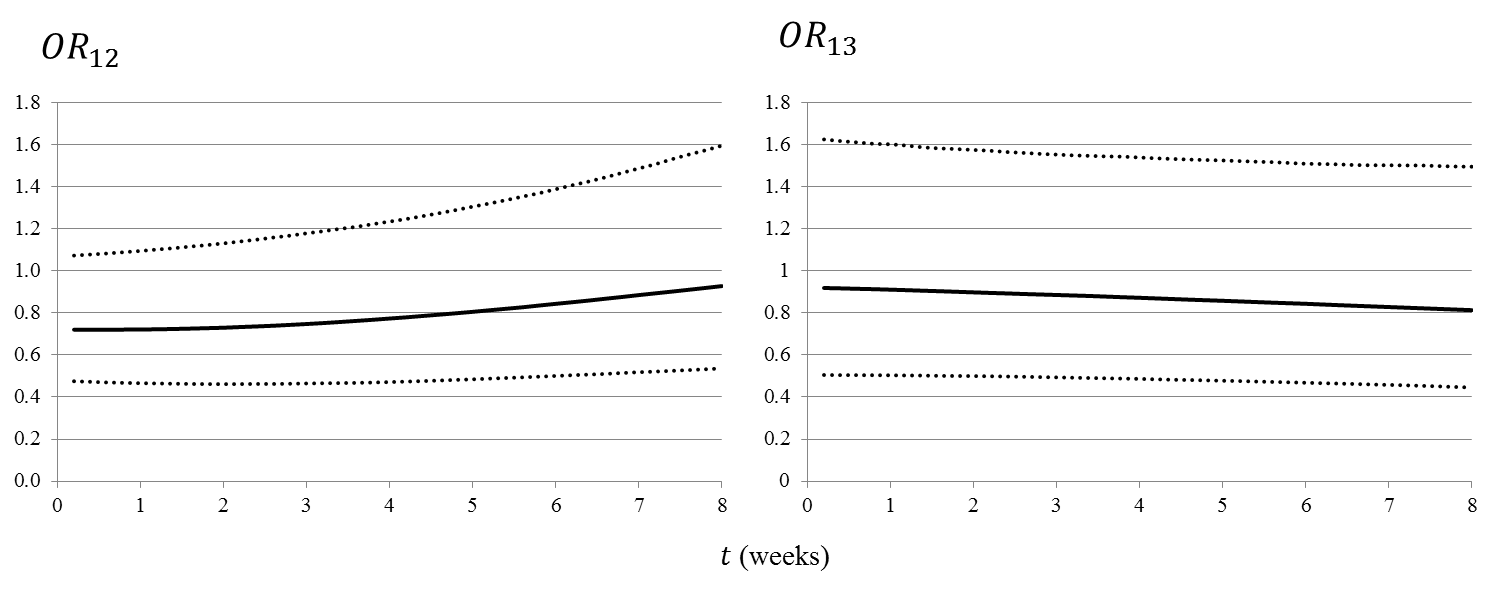


# Mathematical details of the 4-state model

Here we give exact formulas for the probabilities of a patient being in each of the unobserved states for the 4-state model described in Section 3.6 of the main paper. To simplify notation we suppress the index for the treatment received, $T_{k}$, in all formulas. The following analysis corresponds to the most general case of a four-state model discussed in the main document, i.e. Panel d of Figure 1.

The infinitesimal probability of a patient starting at state 1 at time $t=0$, to enter state 4 between times $t_{D}$ and $t_{D}+dt_{D}$ is $\pi_{1,1}\left( t_{D} \right)\gamma_{14}dt_{D}+\pi_{1,2}\left( t_{D} \right)\gamma_{24}dt_{D}$. After time $\Delta t$ the patient may still be at state 4 with a probability $\pi_{4,4}\left( \Delta t \right)=\frac{\gamma_{54}+\gamma_{45}e^{-\left( \gamma_{45}+\gamma_{54} \right)\Delta t}}{\gamma_{45}+\gamma_{54}}$ [1] or may be at state 5 with probability $\pi_{4,5}\left( \Delta t \right)=1-\pi_{4,4}\left( \Delta t \right)$. Similarly, the infinitesimal probability of a patient entering state 5 between times $t_{D}$ and $t_{D}+dt_{D}$ is $\pi_{1,1}\left( t_{D} \right) \gamma_{15}dt_{D}+\pi_{1,2}\left( t_{D} \right)\gamma_{25}dt_{D}$. After time $\Delta t$ the patient will be at state 5 with a probability $\pi_{5,5}\left( \Delta t \right)=\frac{\gamma_{45}+\gamma_{54}e^{-\left( \gamma_{45}+\gamma_{54} \right)\Delta t}}{\gamma_{45}+\gamma_{54}}$ and at state 4 with probability $\pi_{5,4}\left( \Delta t \right)=1-\pi_{5,5}\left( \Delta t \right)$ [1].

Using these quantities one can estimate the probability of a patient randomized in treatment arm $i$ to be in state 4 (*unobserved non-response*) in time $t$ as:

$$\pi_{1,4}\left( t \right)=\int_{t_{D}=0}^{t} ((\pi_{1,1}\left( t_{D} \right) \gamma_{14}+\pi_{1,2}\left( t_{D} \right)\gamma_{24})\pi_{4,4}\left( t-t_{D} \right)+\left( \pi_{1,1}\left( t_{D} \right)\gamma_{15}+\pi_{1,2}\left( t_{D} \right) \gamma_{25} \right)\pi_{5,4}\left( t-t_{D} \right))dt_{D}$$

The overall probability of non-response, observed or unobserved, is $\pi_{1,1}\left( t \right)+\pi_{1,4}\left( t \right)$.

Similarly, the probability of a patient being in state 5 at time $t$after the start of the study is given by:

$$\pi_{1,5}\left( t \right)=\int_{t_{D}=0}^{t} ((\pi_{1,1}\left( t_{D} \right) \gamma_{14}+\pi_{1,2}\left( t_{D} \right)\gamma_{24})\pi_{4,5}\left( t-t_{D} \right)+\left( \pi_{1,1}\left( t_{D} \right)\gamma_{15}+\pi_{1,2}\left( t_{D} \right) \gamma_{25} \right)\pi_{5,5}\left( t-t_{D} \right))dt_{D}$$

The exact expression for the latter is given below:

$$\pi_{1,5}\left( t \right)=-\frac{1}{\left( \gamma_{45}+\gamma_{54} \right)h}\gamma_{14}\gamma_{45}\left( 1+\frac{h-\lambda_{1}+\lambda_{2}}{h-\lambda_{1}-\lambda_{2}}-\frac{2\left( h+\lambda_{1} \right)}{h+\lambda_{1}+\lambda_{2}}+\frac{4ⅇ^{-\left( \gamma_{45}+\gamma_{54} \right)t}h\left( \gamma_{45}+\gamma_{54}-\lambda_{2} \right)}{\left( {2\gamma}_{45}+{2\gamma}_{54}+h-\lambda_{1}-\lambda_{2} \right)\left( -2\gamma_{45}-2\gamma_{54}+h+\lambda_{1}+\lambda_{2} \right)}+2ⅇ^{-\frac{1}{2}t\left( h+\lambda1+\lambda2 \right)}\left( \gamma_{45}+\gamma_{54} \right)\left( -\frac{ⅇ^{ht}\left( h-\lambda_{1}+\lambda_{2} \right)}{\left( h-\lambda_{1}-\lambda_{2} \right)\left( 2\gamma_{45}+2\gamma_{54}+h-\lambda_{1}-\lambda_{2} \right)}-\frac{h+\lambda_{1}-\lambda_{2}}{\left( h+\lambda_{1}+\lambda2 \right)\left( -2\gamma_{45}-2\gamma_{54}+h+\lambda_{1}+\lambda_{2} \right)} \right) \right)+\frac{1}{4\left( \gamma_{45}+\gamma_{54} \right)h\gamma_{21}}\gamma_{25}\left( h+\lambda1-\lambda2 \right)\left( h-\lambda_{1}+\lambda_{2} \right)\left( 2ⅇ^{-\frac{1}{2}t\left( h+\lambda1+\lambda2 \right)}\left( ⅇ^{ht}\left( \frac{\gamma_{45}}{h-\lambda_{1}-\lambda_{2}}+\frac{\gamma_{54}}{{2\gamma}_{45}+2\gamma_{54}+h-\lambda_{1}-\lambda_{2}} \right)+\frac{\gamma_{45}}{h+\lambda_{1}+\lambda_{2}}+\frac{\gamma_{54}}{{-2\gamma}_{45}-2\gamma_{54}+h+\lambda_{1}+\lambda_{2}} \right)-2\left( \frac{2ⅇ^{-\left( \gamma_{45}+\gamma_{54} \right)t}\gamma_{54}h}{\left( {2\gamma}_{45}+{2\gamma}_{54}+h-\lambda_{1}-\lambda_{2} \right)\left( -{2\gamma}_{45}-2\gamma_{54}+h+\lambda_{1}+\lambda_{2} \right)}+\gamma_{45}\left( \frac{1}{h-\lambda_{1}-\lambda_{2}}+\frac{1}{h+\lambda_{1}+\lambda_{2}} \right) \right) \right)+\frac{1}{2h(\gamma_{45}+\gamma_{545s given below:ssion for the latter})}\gamma_{15}$$

$$(-\frac{2\gamma_{45}\left( h-\lambda_{1}+\lambda_{2} \right)}{h-\lambda_{1}-\lambda_{2}}+\frac{2\gamma_{45}\left( h+\lambda_{1}-\lambda_{2} \right)}{h+\lambda_{1}+\lambda_{2}}+\frac{8ⅇ^{-t\left( \gamma_{45}+\gamma_{54} \right)}h\gamma_{54}\left( \gamma_{45}+\gamma_{54}-\lambda2 \right)}{\left( h+2\gamma_{45}+2\gamma_{54}-\lambda_{1}-\lambda_{2} \right)\left( h-2\gamma_{45}-2\gamma_{54}+\lambda_{1}+\lambda_{2} \right)}+2ⅇ^{-\frac{1}{2}t\left( h+\lambda_{1}+\lambda_{2} \right)}(\gamma_{45}+\gamma_{54})(\frac{ⅇ^{ht}(h+2\gamma_{45}-\lambda_{1}-\lambda_{2})(h-\lambda_{1}+\lambda_{2})}{(h-\lambda_{1}-\lambda_{2})(h+2\gamma_{45}+2\gamma_{54}-\lambda_{1}-\lambda_{2})}-\frac{(h+\lambda_{1}-\lambda_{2})(h-2\gamma_{45}+\lambda_{1}+\lambda_{2})}{(h+\lambda_{1}+\lambda_{2})(h-2\gamma_{45}-2\gamma_{54}+\lambda_{1}+\lambda_{2})}))$$

$$-\frac{1}{2h\gamma_{21}(\gamma_{45}+\gamma_{54})}\gamma_{24}\gamma_{45}(h+\lambda_{1}-\lambda_{2})(h-\lambda_{1}+\lambda_{2})(\frac{1}{h-\lambda_{1}-\lambda_{2}}+\frac{1}{h+\lambda_{1}+\lambda_{2}}-\frac{2ⅇ^{-t(\gamma_{45}+\gamma_{54})}h}{(h+2\gamma_{45}+2\gamma_{54}-\lambda_{1}-\lambda_{2})(h-2\gamma_{45}-2\gamma_{54}+\lambda_{1}+\lambda_{2})}+ⅇ^{-\frac{1}{2}t(h+\lambda_{1}+\lambda_{2})}(-\frac{1}{h+\lambda_{1}+\lambda_{2}}+\frac{1}{h-2\gamma_{45}-2\gamma_{54}+\lambda_{1}+\lambda_{2}}+ⅇ^{ht}(\frac{1}{h+2\gamma_{45}+2\gamma_{54}-\lambda_{1}-\lambda_{2}}+\frac{1}{-h+\lambda_{1}+\lambda_{2}})))$$

Note that $\pi_{1,4}\left( t \right)=1-\pi_{1,1}\left( t \right)-\pi_{1,2}\left( t \right)-\pi_{1,5}\left( t \right)$.The overall probability of response, either observed or unobserved, is $\pi_{1,2}\left( t \right)+\pi_{1,5}\left( t \right)$.

As an illustration, in Figure 3 we present the probability of either observed or unobserved response, estimated using the four-state model under a MAR assumption (Scenario ii, Section 3.6 of the main paper) for the schizophrenia example.

*Figure 3: Probability of either observed or unobserved non-response (left panel) and probability of either observed or unobserved response (right panel) under the MAR assumption. Βlue for Amisulpride, red for Risperidone*


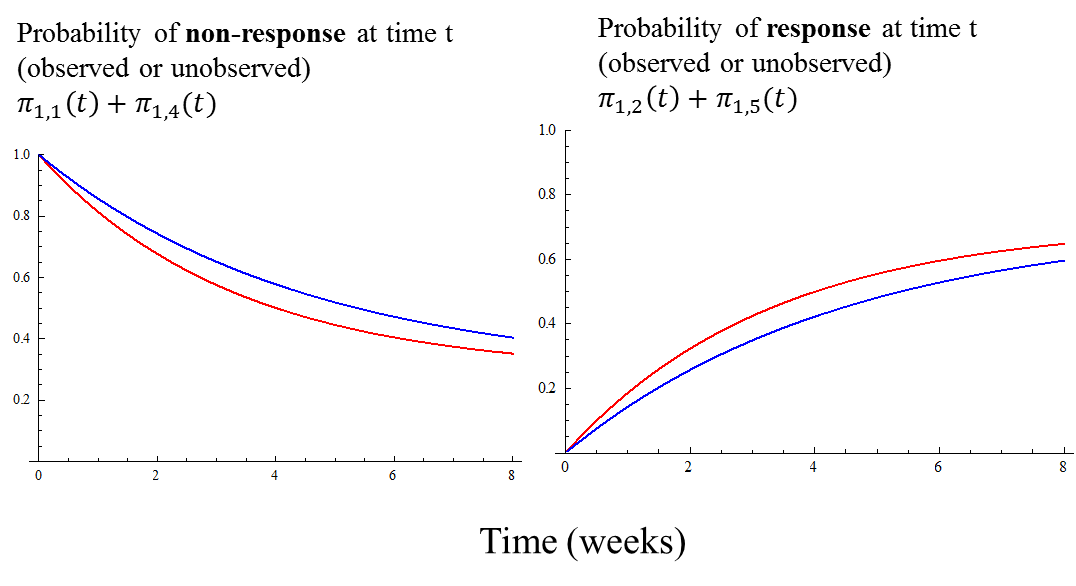


**References**

1. Welton NJ, Ades AE: Estimation of Markov Chain Transition Probabilities and Rates from Fully and Partially Observed Data: Uncertainty Propagation, Evidence Synthesis, and Model Calibration. *Med Decis Making* 2005, 25:633–645.

# OpenBUGS code

The data needed as input are:

npatients: the total number of patients in the study. In our example 228.

numberofobs: the number of observations on each patient. In our example 6.

time: the vector with the time points for each observation, time=c(1,2,3,4,6,8)

obs: the observations for each patient coded as 1 (non-response), 2 (response), 3 (dropout). This is a matrix with dimensions (npatients)$\times$(numberofobs)

treat: a vector with dimension equal to the number of patients, encoding the treatment administered to each patient as either 1 or 2.

The code used is:

model{

### calculating time between observations

for (j in 2:numberofobs){Dt[j]<-time[j] - time[j-1]}

Dt[1]<-time[1]

### calculating λ’s and h

for (i in 1:npatients){

lambda1[i]<-GG12[i]+GG13[i]

lambda2[i]<-GG21[i]+GG23[i]

h[i]<-sqrt(pow(lambda1[i]-lambda2[i], 2) + 4*GG12[i]*GG21[i])

### Likelihood of the model

for (j in 1:numberofobs){

x[i,j,1]<-equals(1,obs[i,j])

x[i,j,2]<-equals(2,obs[i,j])

x[i,j,3]<-equals(3,obs[i,j])}

for (j in 2:numberofobs){

p[i,j,1]<-Pi11[i,j]*equals(1,obs[i,j-1])+Pi21[i,j]*equals(2,obs[i,j-1])

p[i,j,2]<-Pi12[i,j]*equals(1,obs[i,j-1])+Pi22[i,j]*equals(2,obs[i,j-1])

p[i,j,3]<-Pi13[i,j]*equals(1,obs[i,j-1])+Pi23[i,j]*equals(2,obs[i,j-1])+equals(3,obs[i,j-1])}

p[i,1,1]<-Pi11[i,1]

p[i,1,2]<-Pi12[i,1]

p[i,1,3]<-Pi13[i,1]

for (j in 1:numberofobs){x[i,j,1:3]~dmulti(p[i,j,1:3],1)}

for (j in 1:numberofobs){

e1[i,j]<-exp(-0.5*(lambda1[i]+lambda2[i]-h[i])*Dt[j])

e2[i,j]<-exp(-0.5*(lambda1[i]+lambda2[i]+h[i])*Dt[j])

Pi11[i,j]<-((-lambda1[i]+lambda2[i]+h[i])*e1[i,j]+(lambda1[i]-lambda2[i]+h[i])*e2[i,j])/(2*h[i])

Pi12[i,j]<-(-lambda1[i]+lambda2[i]+h[i])*(lambda1[i]-lambda2[i]+h[i])*(e1[i,j]-e2[i,j])/(4*h[i]*GG21[i])

Pi13[i,j]<-1-Pi11[i,j]-Pi12[i,j]

Pi21[i,j]<-GG21[i]*(e1[i,j]-e2[i,j])/h[i]

Pi22[i,j]<-((lambda1[i]-lambda2[i]+h[i])*e1[i,j]+(-lambda1[i]+lambda2[i]+h[i])*e2[i,j])/(2*h[i])

Pi23[i,j]<-1-Pi21[i,j]-Pi22[i,j]}}

#### Prior distributions

log(G12[difftreat[1]])<-u12[difftreat[1]]

log(G12[difftreat[2]])<-u12[difftreat[2]]

u12[difftreat[1]]~ dunif(-10,5)

u12[difftreat[2]]~ dunif(-10,5)

theta12<-u12[difftreat[1]]-u12[difftreat[2]]

log(G21[difftreat[1]])<-u21[difftreat[1]]

log(G21[difftreat[2]])<-u21[difftreat[2]]

u21[difftreat[1]]~ dunif(-10,5)

u21[difftreat[2]]~ dunif(-10,5)

theta21<-u21[difftreat[1]]-u21[difftreat[2]]

log(G23[difftreat[1]])<-u23[difftreat[1]]

log(G23[difftreat[2]])<-u23[difftreat[2]]

u23[difftreat[1]]~ dunif(-10,5)

u23[difftreat[2]]~ dunif(-10,5)

theta23<-u23[difftreat[1]]-u23[difftreat[2]]

log(G13[difftreat[1]])<-u13[difftreat[1]]

log(G13[difftreat[2]])<-u13[difftreat[2]]

u13[difftreat[1]]~ dunif(-10,5)

u13[difftreat[2]]~ dunif(-10,5)

theta13<-u13[difftreat[1]]-u13[difftreat[2]]

## Accounting for patient-level heterogeneity in the transition rates

for (i in 1:npatients){

log(GG12[i])<-uu12[i]

uu12[i]~dnorm(u12[treat[i]],precg)

log(GG21[i])<-uu21[i]

uu21[i]~dnorm(u21[treat[i]],precg)

log(GG13[i])<-uu13[i]

uu13[i]~dnorm(u13[treat[i]],precg)

log(GG23[i])<-uu23[i]

uu23[i]~dnorm(u23[treat[i]],precg)

}

precg<-1/tg.s

tg.s<-tg*tg ### common heterogeneity

tg~dunif(0,1)

TF<-time[numberofobs] ### the study’s endpoint

Dta<-8 ### this is the time increment

for (j in 1:1){T[j]<-Dta*j} ### increase j if you want to obtain estimates for more time points

for (i in 1:2){

tlambda1[i]<-G12[i]+G13[i]

tlambda2[i]<-G21[i]+G23[i]

th[i]<-sqrt(pow(tlambda1[i]-tlambda2[i], 2) + 4*G12[i]*G21[i])

G45[i]<-0.1*G12[i] ### for the 4-state model (MNAR)

G54[i]<-2*G21[i]

G14[i]<-0.9*G13[i]

G15[i]<-0.1*G13[i]

G24[i]<-0.9*G23[i]

G25[i]<-0.1*G23[i]

for (j in 1:1){

Ae1[i,j]<-exp(-0.5*(tlambda1[i]+tlambda2[i]-th[i])*T[j])

Ae2[i,j]<-exp(-0.5*(tlambda1[i]+tlambda2[i]+th[i])*T[j])

APi11[i,j]<-((-tlambda1[i]+tlambda2[i]+th[i])*Ae1[i,j]+(tlambda1[i]-tlambda2[i]+th[i])*Ae2[i,j])/(2*th[i])

APi12[i,j]<-(-tlambda1[i]+tlambda2[i]+th[i])*(tlambda1[i]-tlambda2[i]+th[i])*(Ae1[i,j]-Ae2[i,j])/(4*th[i]*G21[i])

APi13[i,j]<-1-APi11[i,j]-APi12[i,j]

APi21[i,j]<-G21[i]*(Ae1[i,j]-Ae2[i,j])/th[i]

APi22[i,j]<-((tlambda1[i]-tlambda2[i]+th[i])*Ae1[i,j]+(-tlambda1[i]+tlambda2[i]+th[i])*Ae2[i,j])/(2*th[i])

APi23[i,j]<-1-APi21[i,j]-APi22[i,j]

APi13D[i,j]<-G13[i]/(G12[i]+G13[i])*(1-exp(-( G12[i]+G13[i])*T[j]))

APi123[i,j]<-G23[i]*(-tlambda1[i]+tlambda2[i]+th[i])*(tlambda1[i]-tlambda2[i]+th[i])*

(1/(tlambda1[i]+tlambda2[i]-th[i])-1/(tlambda1[i]+tlambda2[i]+th[i])+

exp(-0.5*T[j]*(tlambda1[i]+tlambda2[i]-th[i]))/(-tlambda1[i]-tlambda2[i]+th[i])+

exp(-0.5*T[j]*(tlambda1[i]+tlambda2[i]+th[i])) /(tlambda1[i]+tlambda2[i]+th[i]))/(2*th[i]*G21[i])

### The following probabilities correspond to the 4-state model. Calculations for each probability are performed in pieces

AP141a[i,j]<-1 / (2* (G45[i]+G54[i]) * th[i] )* G14[i]*

((-2*G54[i]*(-tlambda1[i]+tlambda2[i]+th[i])

/(-tlambda1[i]-tlambda2[i]+th[i])+

2*G54[i]*(tlambda1[i]-tlambda2[i]+th[i])/(tlambda1[i]+tlambda2[i]+th[i])))

AP142a[i,j]<-1 / (2* (G45[i]+G54[i]) * th[i] )* G14[i]*

((8*exp(-(G54[i]+G45[i])*T[j])*G45[i]*th[i]*(G45[i]+G54[i]-tlambda2[i])/

((2*G45[i]+2*G54[i]+th[i]-tlambda1[i]-tlambda2[i])*(-2*G45[i]-2*G54[i]+th[i]+tlambda1[i]+tlambda2[i]))))

AP143a[i,j]<-

1 / (2* (G45[i]+G54[i]) * th[i] )* G14[i]*

(2*Ae1[i,j]*(G45[i]+G54[i])*(2*G54[i]+th[i]-tlambda1[i]-tlambda2[i])*(th[i]-tlambda1[i]+tlambda2[i])/

((th[i]-tlambda1[i]-tlambda2[i])*(2*G45[i]+2*G54[i]+th[i]-tlambda1[i]-tlambda2[i])))

AP144a[i,j]<--1 / (2* (G45[i]+G54[i]) * th[i] )* G14[i]*

(2*Ae2[i,j]*(G45[i]+G54[i])*(-2*G54[i]+th[i]+tlambda1[i]+tlambda2[i])*(th[i]+tlambda1[i]-tlambda2[i])/

((th[i]+tlambda1[i]+tlambda2[i])*(-2*G45[i]-2*G54[i]+th[i]+tlambda1[i]+tlambda2[i])))

AP14a[i,j]<-AP141a[i,j]+AP142a[i,j]+AP143a[i,j]+AP144a[i,j]}

for (j in 1:1){

factor14b[i,j]<-G54[i]*G25[i]*(th[i]+tlambda1[i]-tlambda2[i])*(th[i]-tlambda1[i]+tlambda2[i])/

(2*(G45[i]+G54[i])*th[i]*G21[i])

AP141b[i,j]<-factor14b[i,j]/(-th[i]+tlambda1[i]+tlambda2[i])

AP142b[i,j]<--factor14b[i,j]/(th[i]+tlambda1[i]+tlambda2[i])

AP143b[i,j]<- factor14b[i,j]*(2*exp(-(G54[i]+G45[i])*T[j])*th[i]/

((2*G45[i]+2*G54[i]+th[i]-tlambda1[i]-tlambda2[i])*(-2*G45[i]-2*G54[i]+th[i]+tlambda1[i]+tlambda2[i])))

AP144b[i,j]<- factor14b[i,j]*

Ae2[i,j]/(th[i]+tlambda1[i]+tlambda2[i])

AP145b[i,j]<- -factor14b[i,j]*

Ae2[i,j]/(-2*G45[i]-2*G54[i]+th[i]+tlambda1[i]+tlambda2[i])

AP146b[i,j]<--factor14b[i,j]*Ae1[i,j]/(2*G45[i]+2*G54[i]+th[i]-tlambda1[i]-tlambda2[i])

AP147b[i,j]<--factor14b[i,j]*Ae1[i,j]/(-th[i]+tlambda1[i]+tlambda2[i])

AP14b[i,j]<-AP141b[i,j]+AP142b[i,j]+AP143b[i,j]+AP144b[i,j]+AP145b[i,j]+AP146b[i,j]+AP147b[i,j]

factor14c[i,j]<--G15[i]*G54[i]/(2*th[i]*(G45[i]+G54[i]))

AP141c[i,j]<-factor14c[i,j]*(2+2*(th[i]-tlambda1[i]+tlambda2[i])/(th[i]-tlambda1[i]-tlambda2[i]))

AP142c[i,j]<-factor14c[i,j]*(-4*(th[i]+tlambda1[i])/(th[i]+tlambda2[i]+tlambda1[i]))

AP143c[i,j]<-factor14c[i,j]*

(

8*exp(-(G54[i]+G45[i])*T[j])*th[i]*(G54[i]+G45[i]-tlambda2[i])/((2*G45[i]+2*G54[i]+th[i]-tlambda1[i]-tlambda2[i])*(-2*G45[i]-2*G54[i]+th[i]+tlambda1[i]+tlambda2[i])))

AP144c[i,j]<-factor14c[i,j]*4*Ae1[i,j]*(G45[i]+G54[i])*

(-(th[i]-tlambda1[i]+tlambda2[i])/

((th[i]-tlambda1[i]-tlambda2[i])*(2*G45[i]+2*G54[i]+th[i]-tlambda1[i]-tlambda2[i])))

AP145c[i,j]<-factor14c[i,j]*4*Ae2[i,j]*(G45[i]+G54[i])*

(-(th[i]+tlambda1[i]-tlambda2[i])/

((th[i]+tlambda1[i]+tlambda2[i])*(-2*G45[i]-2*G54[i]+th[i]+tlambda1[i]+tlambda2[i])))

AP14c[i,j]<- AP141c[i,j]+AP142c[i,j]+AP143c[i,j]+AP144c[i,j]+AP145c[i,j]

factor14d[i,j]<-1/(4*th[i]*G21[i]*(G45[i]+G54[i]))*G24[i]*(th[i]+tlambda1[i]-tlambda2[i])*(th[i]-tlambda1[i]+tlambda2[i])

AP141d[i,j]<-factor14d[i,j]*

(2*Ae2[i,j]*G54[i]/(th[i]+tlambda1[i]+tlambda2[i]))

AP142d[i,j]<-factor14d[i,j]*

(2*Ae2[i,j]*G45[i]/(

-2*G45[i]-2*G54[i]+th[i]+tlambda1[i]+tlambda2[i]

))

AP143d[i,j]<-factor14d[i,j]*

(2*Ae1[i,j]*

(G45[i]/(2*G45[i]+2*G54[i]+th[i]-tlambda1[i]-tlambda2[i])))

AP144d[i,j]<-factor14d[i,j]*

(-2*Ae1[i,j]*G54[i]/(-th[i]+tlambda1[i]+tlambda2[i]))

AP145d[i,j]<-factor14d[i,j]*

(-4*exp(-(G54[i]+G45[i])*T[j])*th[i]*G45[i]

/((2*G45[i]+2*G54[i]+th[i]-tlambda1[i]-tlambda2[i])*(-2*G45[i]-2*G54[i]+th[i]+tlambda1[i]+tlambda2[i])))

AP146d[i,j]<-factor14d[i,j]*

(-2*G54[i]*(1/(th[i]-tlambda1[i]-tlambda2[i])+1/(th[i]+tlambda1[i]+tlambda2[i])))

AP14d[i,j]<-AP141d[i,j]+AP142d[i,j]+AP143d[i,j]+AP144d[i,j]+AP145d[i,j]+AP146d[i,j]

AP14[i,j]<-AP14a[i,j]+AP14b[i,j]+AP14c[i,j]+AP14d[i,j]

APnonresponseALL[i,j]<-AP14[i,j]+APi11[i,j]

APresponseALL[i,j]<-1-APnonresponseALL[i,j]}}

### Odds ratios

for (j in 1:1){

OR12[j]<-APi12[2,j]*(1-APi12[1,j])/(APi12[1,j]*(1-APi12[2,j]))

LOR12[j]<-log(OR12[j])

OR21[j]<-APi21[2,j]*(1-APi21[1,j])/(APi21[1,j]*(1-APi21[2,j]))

LOR21[j]<-log(OR21[j])

OR13[j]<-APi13[2,j]*(1-APi13[1,j])/(APi13[1,j]*(1-APi13[2,j]))

LOR13[j]<-log(OR13[j])

OR13D[j]<-APi13D[2,j]*(1-APi13D[1,j])/(APi13D[1,j]*(1-APi13D[2,j]))

LOR13D[j]<-log(OR13D[j])

OR123[j]<-APi123[2,j]*(1-APi123[1,j])/(APi123[1,j]*(1-APi123[2,j]))

LOR123[j]<-log(OR123[j])

OR23[j]<-APi23[2,j]*(1-APi23[1,j])/(APi23[1,j]*(1-APi23[2,j]))

LOR23[j]<-log(OR23[j])

OR12ALL[j]<-APresponseALL[2,j]*(1-APresponseALL[1,j])/(APresponseALL[1,j]*(1-APresponseALL[2,j]))

LOR12ALL[j]<-log(OR12ALL[j])}

##ETS

for (i in 1:2){

ETS1[i]<-(-tlambda1[i]+tlambda2[i]+th[i])/(th[i]*(tlambda1[i]+tlambda2[i]-th[i]))*

(1-exp(-0.5*TF*(tlambda1[i]+tlambda2[i]-th[i])))+

(tlambda1[i]-tlambda2[i]+th[i])/(th[i]*(tlambda1[i]+tlambda2[i]+th[i]))*

(1-exp(-0.5*TF*(tlambda1[i]+tlambda2[i]+th[i])))

ETS2[i]<-(-tlambda1[i]+tlambda2[i]+th[i])*(tlambda1[i]-tlambda2[i]+th[i])*

(1/(tlambda1[i]+tlambda2[i]-th[i])-1/(tlambda1[i]+tlambda2[i]+th[i])+

exp(-0.5*TF*(tlambda1[i]+tlambda2[i]-th[i]))/(-tlambda1[i]-tlambda2[i]+th[i])+

exp(-0.5*TF*(tlambda1[i]+tlambda2[i]+th[i]))/(tlambda1[i]+tlambda2[i]+th[i]))/(2*th[i]*G21[i])

ETS3[i]<-TF-ETS1[i]-ETS2[i]}

dETS1<-ETS1[2]-ETS1[1]

dETS2<-ETS2[2]-ETS2[1]

dETS3<-ETS3[2]-ETS3[1]

}

}}

1. Department of Hygiene and Epidemiology, University of Ioannina School of Medicine, Ioannina, Greece. [↑](#footnote-ref-1)
2. School of Social and Community Medicine, University of Bristol, UK [↑](#footnote-ref-2)
3. Department of Psychiatry and Psychotherapy, Technische Universität München, Klinikum rechts der Isar, Ismaningerstr. 22, 81675 München, Germany.

   ^4^ Institute of Social and Preventive Medicine, University of Bern, Bern, Switzerland [↑](#footnote-ref-3)
